# Measurements of δ^13^C in CH_4_ and using particle dispersion modeling to characterize sources of Arctic methane within an air mass

**DOI:** 10.1002/2016JD026006

**Published:** 2016-12-13

**Authors:** J. L. France, M. Cain, R. E. Fisher, D. Lowry, G. Allen, S. J. O'Shea, S. Illingworth, J. Pyle, N. Warwick, B. T. Jones, M. W. Gallagher, K. Bower, M. Le Breton, C. Percival, J. Muller, A. Welpott, S. Bauguitte, C. George, G. D. Hayman, A. J. Manning, C. Lund Myhre, M. Lanoisellé, E. G. Nisbet

**Affiliations:** ^1^ Department of Earth Sciences, Royal Holloway University of London Egham UK; ^2^ School of Environmental Sciences University of East Anglia Norwich UK; ^3^ National Centre for Atmospheric Science University of Cambridge Cambridge UK; ^4^ School of Earth, Atmospheric and Environmental Sciences University of Manchester Manchester UK; ^5^ Faculty of Science and Engineering Manchester Metropolitan University Manchester UK; ^6^ Facility for Airborne Atmospheric Measurements (FAAM), Building 125 Cranfield University Cranfield UK; ^7^ Centre for Ecology and Hydrology Wallingford UK; ^8^ UK Met Office Exeter UK; ^9^ Department Atmospheric and Climate Research NILU–Norwegian Institute for Air Research Kjeller Norway

**Keywords:** methane, wetlands, Arctic, d13C

## Abstract

A stratified air mass enriched in methane (CH_4_) was sampled at ~600 m to ~2000 m altitude, between the north coast of Norway and Svalbard as part of the Methane in the Arctic: Measurements and Modelling campaign on board the UK's BAe‐146‐301 Atmospheric Research Aircraft. The approach used here, which combines interpretation of multiple tracers with transport modeling, enables better understanding of the emission sources that contribute to the background mixing ratios of CH_4_ in the Arctic. Importantly, it allows constraints to be placed on the location and isotopic bulk signature of the emission source(s). Measurements of δ^13^C in CH_4_ in whole air samples taken while traversing the air mass identified that the source(s) had a strongly depleted bulk δ^13^C CH_4_ isotopic signature of −70 (±2.1)‰. Combined Numerical Atmospheric‐dispersion Modeling Environment and inventory analysis indicates that the air mass was recently in the planetary boundary layer over northwest Russia and the Barents Sea, with the likely dominant source of methane being from wetlands in that region.

## Key Points

- Northern Russian wetlands can contribute notably to methane in the remote Arctic
- Isotopic bulk signature of ^13^CH_4_ determined in air mass with enhanced methane observed in the Arctic
- Isotopic source signature for δ^13^C for CH_4_ bulk input from northern Russian wetlands and potential shallow shelf region offshore is <−70‰

## 1 Introduction

Methane (CH_4_) is well known to be a powerful greenhouse gas, with approximately 28 times the global warming potential of carbon dioxide over a 100 year period, and is the second most important anthropogenic greenhouse gas in terms of radiative forcing [*Denman et al*., 2007; *Myhre et al*., 2013]. The Intergovernmental Panel on Climate Change [*Denman et al*., 2007] has previously highlighted that terrestrial carbon flux processes are complex with high uncertainties and that continued investigation to understand the role of CH_4_ in the atmosphere is vital. It is especially important to understand CH_4_ sources in the Arctic as temperatures there are rising twice as fast as global averages and are expected to continue to rise [*Parmentier et al*., 2013]. The increasing temperatures could destabilize reservoirs of CH_4_ from terrestrial and oceanic permafrost and marine hydrates [*O'Connor et al*., 2010], as well as leading to increased fluxes from Arctic wetlands.

Sources of CH_4_ to the Arctic are dominated in summer by wetland emissions [*Kirschke et al*., 2013]. Wetlands globally provide a CH_4_ flux to the atmosphere of 142–208 Tg yr^−1^ out of a total of ~550 Tg yr^−1^ from all CH_4_ sources, but for the Arctic budget it is less clear, as recent work has struggled to constrain the wetland contributions spatially due to inconsistencies comparing ground mapping and remote sensing of wetlands [*Melton et al*., 2013]. Older estimates put the total emission from wetlands above 50°N as 10–15% of the total global wetland contribution [*Christensen et al*., 1996]. Other sources of CH_4_ within the Arctic include the tundra permafrost melt [*Wille et al*., 2008], subsea permafrost and hydrate degradation [*Shakhova et al*., 2014; *Vonk et al*., 2012; *Westbrook et al*., 2009], Arctic ocean surface waters [*Kort et al*., 2012], natural geological CH_4_ seepage [*Walter et al*., 2012], and anthropogenic emissions such as fugitive emissions from oil and gas platforms. Sources such as CH_4_ hydrates are not as yet thought to be contributing significantly to the Arctic CH_4_ budget (e.g., *Kirschke et al*. [2013] attribute 6 Tg yr^−1^ globally from hydrates), but it has been suggested that they might do so more significantly in the future with increasing temperatures [*Biastoch et al*., 2011]. Removal of CH_4_ from the atmosphere is dominated by oxidation through tropospheric OH radical interaction (~85%) [*Lelieveld et al*., 1998].

Recently, the global CH_4_ budget has been seen to be changing with year‐to‐year increases since 2007 [*Nisbet et al*., 2014; *Sussmann et al*., 2012]. The cause of this increase is thought to be dominated by changes to the tropical wetland emissions [*Bousquet et al*., 2011; *Nisbet et al*., 2014] or agricultural activities (ruminants and rice cultivation [*Schaefer et al*., 2016]), with the drivers for the tropical wetland growth thought to be a combination of high precipitation and high temperatures, enhancing biogenic activity [*Dlugokencky et al*., 2009]. As many of the sources of Arctic CH_4_ are at least partly temperature dependent, the projected Arctic temperature rise of between 2.2°C and 8.3°C by 2100 [*Collins et al*., 2013] makes an urgent case for better understanding of Arctic CH_4_ and the effect of temperature rises on sources of CH_4_ emissions. Recent studies have identified previously unquantified sources of atmospheric CH_4_ to the Arctic, such as subsea permafrost degradation [*Portnov et al*., 2014; *Portnov et al*., 2013], CH_4_ bubbling, and geologically old CH_4_ seepage along thaw features [*Walter et al*., 2008, 2012], all of which have been linked to the increasing Arctic temperatures.

**Figure 1:**
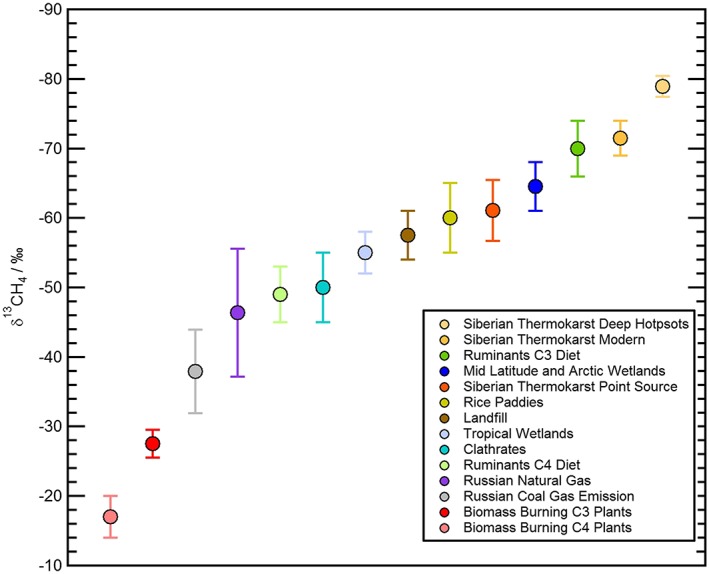
Isotopic ranges of δ^13^C for CH_4_ for a variety of CH_4_ sources. The data for the graph and the corresponding uncertainties use data from *Bergamaschi et al*. [1998], *Cramer et al*. [1999], *Dlugokencky et al*. [2011], *Fisher et al*. [2011], *Lowry et al*. [2001], *Monteil et al*. [2011], *Sherwood et al*. [2016], *Sriskantharajah et al*. [2012], *Umezawa et al*. [2011], *Walter et al*. [2008], and *Zhang et al*. [2013].

The ratio of ^13^C:^12^C (expressed relative to the Pee Dee belemnite standard as δ^13^C) in CH_4_ (along with the ratio of D:H) can be used to help determine the origin of detected CH_4_ emissions. Light CH_4_ (depleted in ^13^C) is emitted mainly during biological production and isotopically varies depending on the amount of oxidation occurring before emission (e.g., during transport in soil or water); on the other hand, heavy CH_4_ (relatively enriched in ^13^C compared to biological sources) comes from pyrogenic and thermogenic sources such as biomass burning and coal mines [*Zazzeri et al*., 2015]. Figure 1 demonstrates how the δ^13^C value varies between differing sources of CH_4_. Much work has been done at specific localities in order to determine isotopic source signatures for differing sources [e.g., *Fisher et al*., 2006; *Iverach et al*., 2015; *Zazzeri et al*., 2015], and a comprehensive database has been set up to allow much more rigorous selection of δ^13^C values for use in both global and regional modeling studies [*Sherwood et al*., 2016]. Use of the updated δ^13^C database has already demonstrated that commonly used natural gas and coal δ^13^C values in previous top‐down studies have been poorly constrained [*Schwietzke et al*., 2016].

In this work, methane mixing ratios, methane isotope, and supporting measurements from two flights during the Methane in the Arctic: Measurements and Modelling (MAMM) field campaign are combined with air parcel trajectory modeling and previously determined emission inventories. The primary aim is to assess the use of this combination of tools in determining methane emission sources hundreds of kilometers from the measurement location. Previous aircraft campaigns have been undertaken where CH_4_ δ^13^C has been measured in order to determine source characteristics [e.g., *Umezawa et al*., 2011]. However, those flights were performed over expected CH_4_ sources. This current work aims to show the value of determining δ^13^C values for air masses that have an unknown source, by using the δ^13^C signature along with particle dispersion and emission inventory modeling to identify the source(s).

## 2 Methods

### 2.1 Aircraft Measurements

**Figure 2:**
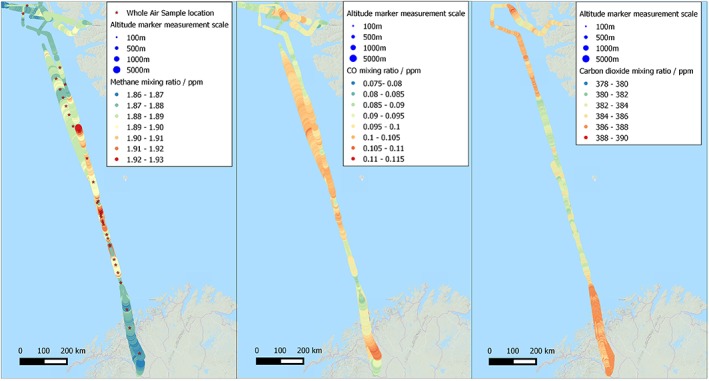
(a) Flight paths and CH_4_ continuous mixing ratio measurements of tropospheric air from the Fast Greenhouse Gas Analyzer (FGGA) and Whole Air Sample (WAS) sampling locations (red stars) for both flights B718 and B719 during the MAMM campaign on 21 July 2012. (b) CO continuous mixing ratio measurements of tropospheric air from the AL5002 UV fluorescence monitor. (c) CO_2_ continuous mixing ratio measurements of tropospheric air from the FGGA. For all plots the variable size of the mixing ratio markers is a reflection of the altitude of the aircraft.

The MAMM program was designed to investigate Arctic CH_4_ using a combination of aircraft and ground measurement studies and complementary modeling approaches. An initial field campaign took place during July 2012 when the Facility for Airborne Atmospheric Measurements (FAAM) modified BAe‐146 Atmospheric Research Aircraft (ARA) was deployed to Kiruna, Sweden (67.850°N, 20.216°E). Seven flights took place over a 4 day period both to survey Arctic wetland areas (see *O'Shea et al*. [2014] for general details of the MAMM campaign) and to measure long‐range transport of CH_4_. The measurements presented here are from two flights on 21 July 2012 (B718 and B719), outbound from Kiruna to and returning from Longyearbyen, Spitsbergen (78.220°N, 15.650°E), respectively. Flight paths for B718 and B719 are shown in Figure 2.

On board the aircraft, and operating continuously, were a Fast Greenhouse Gas Analyzer (FGGA) and a mobile Chemical Ionization Mass Spectrometer (CIMS) whose hydrogen cyanide (HCN) measurements can be used as a tracer for biomass burning [*Tereszchuk et al*., 2011]. The FGGA (model 907‐0010, Los Gatos Research Inc., USA) measures CO_2_, CH_4_, and H_2_O dry air molar fractions using cavity‐enhanced laser absorption spectroscopy at a rate of 1 Hz, with these data available in real time during the flight. The CIMS was used to determine formic acid (HCOOH) and HCN concentrations at a rate of 1 Hz, averaged to 30 s data. In‐flight repeatability was determined using in‐flight gas standards as detailed in *O'Shea et al*. [2013]; for CO_2_ repeatability was determined to be ±0.17 ppm, and typical 1 Hz precision is ±0.66 ppm (all precisions are 1σ). CH_4_ repeatability was determined to be ±1.28 ppb; 1 Hz precision is ±2.48 ppb. For a detailed system description, see *O'Shea et al*. [2013]. In‐flight calibrations of HCOOH were used to determine a relative HCN sensitivity average of 0.4 (±0.01) ion counts s^−1^ ppt^−1^, with a 3σ limit of detection for HCN of 62 ppt (see *Le Breton et al.* [2013] for further details). Carbon monoxide (CO) and ozone (O_3_) measurements were made at 1 Hz using an AL5002 UV fluorescence monitor [*Gerbig et al*., 1999] and a TECO 49C UV photometer, respectively [*Real et al*., 2007]. Wind speed was measured on board the aircraft using the five‐port pressure measurement system, along with static pressure ports and the inertial navigation unit system, providing wind velocity components at 32 Hz, which have been averaged to 1 Hz for this study. For a previous campaign, *Petersen et al*. [2009] estimated the overall uncertainty in horizontal wind measurements to be <±0.5 ms^−1^.

The data from the FGGA were used in real time for decisions on changes to the flight path of the aircraft to optimize sampling and also used to pick appropriate sampling times to fill Whole Air Sample (WAS) steel canisters for further analysis postcampaign. A total of 34 WAS samples were taken in or around the region of enhanced methane. Comparisons of WAS sample measurements in the lab and corresponding FGGA in‐flight measurements show a standard deviation of 2 ppb for CH_4_ and 0.9 ppm for CO_2_ and are normally distributed around the FGGA measurements indicating no systematic bias from the postflight sampling or storage of the WAS samples. Once it was established that the aircraft was flying within the CH_4_‐enhanced air mass, the altitude of the aircraft was varied in order to determine the vertical extent of the air mass and also to map the mixing ratio of CH_4_ throughout. In order for the Keeling analysis method [*Keeling*, 1958, 1961; *Pataki et al*., 2003] to give the best possible precision in determining the isotopic signature of the excess methane in the air mass, the largest possible range of mixing ratios of CH_4_ within the air mass is required. Depending upon the altitude of the aircraft the filling time had to be altered to make the total pressure in the WAS bottles ~300 kPa. The WASs were filled for between 15 s at very low level flying at approximately 100 ft (~30 m) above sea level and for 40 s at 10,000 ft (~3000 m) altitude. The locations where WAS samples were collected are shown in Figure 2a and marked with a red star.

The WAS bottles were returned to the Royal Holloway, University of London greenhouse gas laboratory for postcampaign analysis. CO_2_ and CH_4_ mixing ratios in each WAS sample were measured using a Picarro 1301 cavity ringdown spectroscopic greenhouse gas analyzer for 360 s, with a 180 s flush and 180 s measurement period. During the measurement period the sample was analyzed every 5 s, with an average value determined for the 3 min period. The 1σ precision on the measurements was better than ±0.3 ppb for CH_4_, with small variations between samples. The resulting mixing ratios were corrected for water vapor using the adjustment shown in equation (1).

(1) $${CH}_{4\;\text{dry}}=\;{CH}_{4\;\text{wet}}\;\times \;\left(1+\left(0.01010244\;\times \;H_{2}O\left(\%\right)\right)\right)\text{.}$$

This water vapor correction, which is valid for up to 1.5% H_2_O, was determined using a similar method to that described in *Chen et al*. [2010]. The Picarro 1301 greenhouse gas analyzer is calibrated weekly to the National Oceanic and Atmospheric Administration (NOAA) scale using air standards supplied by Max‐Planck‐Institut, Jena as part of the IMECC (Infrastructure for Measurements of the European Carbon Cycle) project. The Picarro measurements use approximately 1.5 L of air for each WAS bottle measurement routine.

Subsequently, the remaining air in each WAS bottle was analyzed for δ^13^C in CH_4_ using the continuous flow gas chromatography/isotope ratio mass spectrometry method outlined in *Fisher et al*. [2006]. Each WAS bottle was analyzed at least 3 times with a mean repeatability (1σ) of 0.05‰ for δ^13^C in CH_4_.

### 2.2 Particle Dispersion Modeling

The Numerical Atmospheric‐dispersion Modeling Environment (NAME) is a 3‐D Lagrangian particle dispersion model [*Jones et al*., 2007; *Ryall and Maryon*, 1998], which is run here using the UK Meteorological Office's Unified Model meteorological fields [*Cullen*, 1993]. In this study, particles are released from the locations of the WAS samples along flights B718 and B719, with the model then calculating the trajectories of the particles backward in time. The particle motions are calculated based on the large‐scale winds, wind meander, and subgrid‐scale turbulence. NAME has previously been used to identify CH_4_ sources from measurements at Mace Head [*Manning et al*., 2011] and to identify the long‐range transport of biomass burning emissions from Russia to the UK [*Witham and Manning*, 2007], over similar distance scales as to those presented here.

To model the back trajectories from each WAS, 33,333 particles were released at the time and location of the aircraft at the start of each WAS sample. To account for some uncertainty in the model, the particles were released not from a point but from a cube of side 100 m and for 2 min in time (centered on the WAS location). The particles were tracked for 10 days back in time, and the time that the particles spent in the planetary boundary layer of the model was recorded. This information was used to construct the “footprint” maps, which indicate where, during the previous 10 days, air from the surface could have been incorporated into the measured air mass.

## 3 Results and Discussions

### 3.1 Aircraft Observations

**Figure 3:**
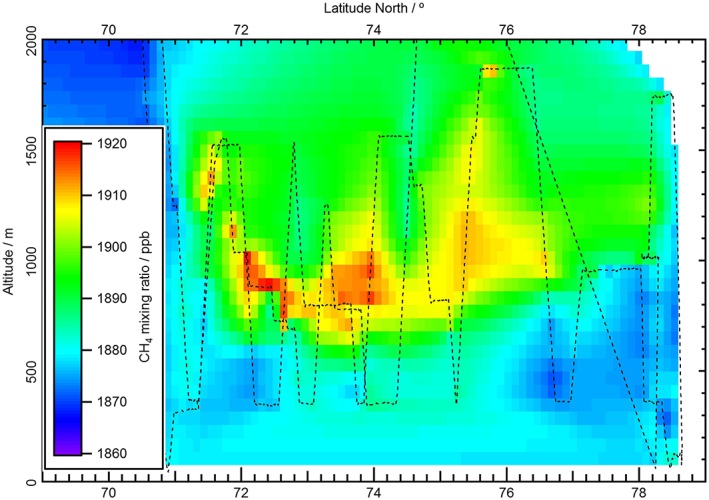
Matrix of linearly interpolated mixing ratios of CH_4_ using the combination of all 1 Hz FGGA data from flights B718 and B719. The flight track is shown in black dashed lines, and the stability of the CH_4_‐enriched air mass can be inferred from the continuity of the interpolated plot at repeated measurement points. The first point of contact with enhanced methane was at 10:15 local time and the final contact at 18:00.

The flight tracks for the two flights, B718 and B719, are shown geographically in Figure 2. Figure 3 contains the combined flight tracks with respect to latitude and elevation so that the profiles of the two flights can be seen. The air mass containing elevated CH_4_ mixing ratios can clearly be visualized by creating a linearly interpolated matrix of continuous CH_4_ mixing ratio measurements plotted against the latitude and altitude as shown in Figure 3. The elevated mixing ratios of CH_4_ were observed between 71.1°N, 17.9°E and 76.5°N, 12.4°E, a distance of ~600 km. A maximum mixing ratio of CH_4_ of ~1920 ppb was observed in the core of the air mass with enhanced methane. The wind speed averaged 8.5 ms^−1^ during the outbound flight and 10 ms^−1^ on the return flight with a predominant easterly/east‐northeasterly wind direction through the sampled region (Figure S1 in the supporting information).

CO and CO_2_ mixing ratios, both made at 1 Hz frequency, are shown in Figures 2b and 2c, respectively, along with scatterplots shown in Figure S2. The CO_2_ measurements display anticorrelation to CH_4_ with minimum CO_2_ associated with high CH_4_ but show very little variation with only a few parts per million change across hundreds of kilometers. The CO measurements are bimodal when plotted against the corresponding CH_4_ measurements, with very low CO mixing ratios associated with the background air and some enhancement of CO at altitudes higher than a few hundred meters above sea level associated with enhanced CH_4_. The presence of higher CO with enhanced CH_4_ could be indicative of a mixed source or simply transportation of long‐lived, CO‐enhanced air from lower latitudes. Polluted air masses from Europe with clear enhancements in both CO_2_ and CO have previously been recorded at Zeppelin Observatory, Svalbard [*Stohl et al*., 2007].

HCN was measured at 1 Hz frequency for flights B718 and B719, as illustrated in Figure S3. HCN is an effective tracer for biomass burning due to its limited sources and its relatively long atmospheric lifetime [*Le Breton et al*., 2013; *Lobert et al*., 1990]. Average HCN mixing ratios were relatively low for flights B718 and B719 at 36.0 (±12.7) ppt and 86.9 (±15.3) ppt, respectively, which are characteristic of expected background concentrations for this region. Furthermore, the observed HCN concentrations display no correlation with measured CH_4_ enhancements, adding evidence that biomass burning did not contribute to the air mass.

The near‐surface O_3_ measured during the two flights shows concentrations in line with those expected from background Arctic surface O_3_ of ~30 ppb, with a gradual increase in concentration with altitude, consistent with a descending and stably stratified free tropospheric air mass. The measurements suggest a lack of recent O_3_ formation above background levels and therefore little input from anthropogenic sources into the air mass.

### 3.2 Associated Measurements from Zeppelin Observatory

Supplementary observations from the Zeppelin Observatory have been explored. The Zeppelin Observatory is a comprehensive atmospheric measurement site located on the west coast of Svalbard, Norway at 78.90°N, 11.88°E on the Zeppelin Mountain, 478 m above sea level. At the Zeppelin Observatory more than 25 greenhouse gases are measured continuously in addition to aerosol variables (optical, physical, and chemical properties) and other atmospheric components (as reactive trace gases). Methane has been measured continuously since 2001 with high time resolution [*Myhre et al*., 2014]. Since 2007 the standard measurement program has been supplemented by taking air samples for δ^13^C in CH_4_ as a part of various research projects and 5 days per week since summer 2012. At Zeppelin, CH_4_ showed elevated levels over a relatively long period during summer 2012 from 17 July to 8 August, but for the days around 21 July, CO, ozone, CO_2_, and sulfate showed mixing ratios typical of Arctic background air.

### 3.3 Using NAME to Identify Potential CH_4_ Source Regions

**Figure 4:**
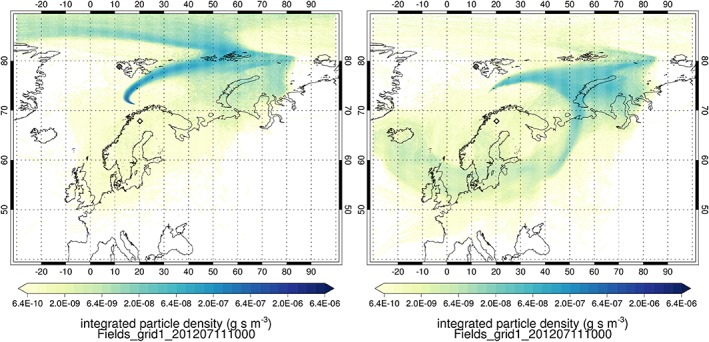
Examples of NAME modeling for flight B718, showing footprint maps from 10 day backward trajectories released from two WAS locations. This shows the modeled interaction with the boundary layer prior to being sampled on board the BAe‐146 (left) WAS flask 4, where CH4 was 1877 ppb. Flask 4 shows the background Arctic air, in contrast to the higher CH_4_ in (right) flask 8, which is coming from parts of Russia and Europe. The diamonds mark the start and end points of B718/B719 at Kiruna and Longyearbyen. In the right plot, particles have been released from WAS flask 8, where CH_4_ was measured at 1912 ppb.

Air mass histories for each of the WAS locations in B718 and B719 have been calculated using the NAME model, as described in section 2.2. Two examples of footprint maps (Figure 4) show where the particles run backward from a particular measurement, passing through the modeled planetary boundary layer. Assuming that the source of the additional CH_4_ in the air mass is emitted from the surface, and that the CH_4_ originated from emissions in the previous 10 days, then the footprint map indicates the weighted source location of the air mass (note that the scale is logarithmic).

The left plot of Figure 4 shows the footprint map generated from particles released at the location of WAS flask 4 during B718 (10:22 UTC; 71.1914°N, 18.5343°E; 963 hPa), where CH_4_ in the flask was 1877 ppb. This is characteristic of background Arctic air, which is supported by the footprint map that shows that the air has come from further north, with the measured air parcel not having been influenced by significant source regions.

The right plot of Figure 4 shows the footprint map from particles released at the location of WAS flask 8 from the same flight (10:58 UTC; 73.1077°N, 16.6945°E, 909 hPa), where CH_4_ was about 35 ppb higher at 1911 ppb. It is clear that, in this case, there is some influence from parts of northern Russia and Europe, including the Pechora River Delta and associated methane‐emitting wetlands. Up to 20% of this region is classified as wetland based on Moderate Resolution Imaging Spectroradiometer (MODIS) land cover maps [*Friedl et al*., 2010]. The measurements could also have been influenced by transport from a large region of the Pechora Sea, Barents Sea, and Kara Sea. The West Yamal Shelf in the Kara Sea has recently been shown to be a likely (and large) source of methane [*Portnov et al*., 2014].

### 3.4 The δ^13^CH_4_ Analysis

The principle behind Keeling plots [*Keeling*, 1958, 1960, 1961] is that the conservation of mass can be applied to an atmospheric system in order to determine source characteristics of a mixed air mass consisting of background air and an added component. If an extra source of CH_4_ is added to a background air mass with a different δ^13^C value, then the overall δ^13^C signature will become a linear combination of the background and the added δ^13^C. At the limit of possibility where the added CH_4_ is effectively infinitely larger than the original concentration then the δ^13^C signature will be entirely from the added CH_4_. The linear extrapolation to the *y* axis of δ^13^C against 1/[CH_4_] will represent the δ^13^C of this infinite mixing ratio of CH_4_ and therefore represents the δ^13^C of the “added (*X*)” CH_4_ component. This is shown mathematically in equations (2) and (3).

(2) $$\delta^{13}C_{\text{measured}}\times \left[\text{measured}\right]=\delta^{13}C_{\text{background}}\times \left[B\right]+\delta^{13}C_{\text{added}}\times \left[X\right]\text{,}$$

(3) $$\delta^{13}C_{\text{measured}}=\left[B\right]\left(\delta^{13}C_{\text{background}}-.\delta^{13}C_{\text{added}}\right)\left(\frac{1}{\left[\text{measured}\right]}\right)+\delta^{13}C_{\text{added}}\text{.}$$

Equations (2) and (3) are modified from *Pataki et al*. [2003]. [*B*] is the mixing ratio of the background CH_4_, [*X*] is the mixing ratio of the added CH_4_, and [measured] is the measured mixing ratio or the sum of [*B*] and [*X*]. The intercept on the *y* axis in equation (3) can be seen to be equal to δ^13^C_added_.

The intercept, and hence the isotopic signature, is found using a linear regression method accounting for individual sample errors and intrinsic scatter in the data using an orthogonal distance regression method to account for errors in both the *x* and *y* axes [*Akritas and Bershady*, 1996].

**Figure 5:**
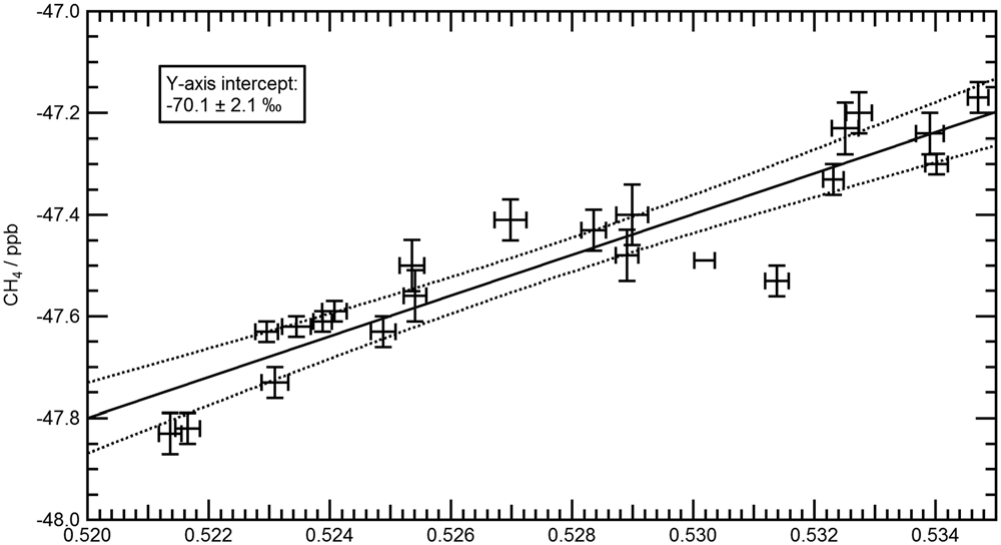
Keeling plot to derive isotopic source signature of the excess CH_4_ over the background mixing ratios. Each point represents a single WAS bottle sample taken during either B718 or B719 flight paths on 21 July 2012. The fitted line is a linear orthogonal regression with fitting errors calculated using variable errors as calculated for each WAS sample. The fitting procedure and error calculation is described in *Akritas and Bershady* [1996].

Measurements of the δ^13^ $C_{{CH}_{4}}$ isotopic signature of CH_4_ provide a powerful constraint in determining emission sources, as discussed earlier and shown in Figure 1. Figure 5 shows the Keeling plot analysis performed on all the WAS samples taken during the two flights in and around the CH_4_‐enhanced air mass. The excess CH_4_ over background has a source δ^13^C signature of −70.1 (±2.1)‰ to 1σ, which is in the range of previously measured δ^13^C signatures of wetland (−68.5 (±0.7)‰ [*Sriskantharajah et al*., 2012]), Eurasian thermokarst lake emissions (−70.3‰ [*Walter et al*., 2008]), and C3 plant‐digesting ruminants (−70 (±4)‰ [*Dlugokencky et al*., 2011]). The δ^13^C value of −70.1 (±2.1)‰ is also consistent with the isotopic signature from other MAMM project flights of based wetland areas in Fennoscandia during the MAMM campaign [*O'Shea et al*., 2014], where values of −70 (±3)‰ were observed at low level above wetlands. However, the regression shown in Figure 5 does in fact display several points slightly offset from the best fit regression line, suggesting that while there is a dominant CH_4_ source there are also some minor and variable additions of CH_4_ from other sources, which are only partially well mixed into the air mass. Given the distance travelled and variable potential sources from the footprint maps in Figure 4, a combination approach using the isotopic information, particle dispersion modeling, and inventory analysis was used to test this hypothesis of multiple sources within the air mass.

### 3.5 NAME Inventory Analysis

The NAME model results show that the air mass histories from the flight track are varied, with influence from both the continent and from polar and oceanic regions. Note that the footprint maps do not include information about where emissions are located, only where the air mass has been within the boundary layer. While sections 3.3 and 3.4 point to the likely dominant source of methane in the air mass measured on 21 July being from Russian wetlands, the NAME analysis on its own does not constrain the emission strength. In this section we explore a further approach to quantification by combining a global CH_4_ emission inventory with the NAME back trajectory analysis.

“Pseudoobservations” have been calculated to assess the contributions from different CH_4_ emission sources in the WAS measurements, using an emission inventory and a transport model. Back trajectories from each of the WAS locations in flights B718 and B719 from the NAME model were used for the transport. The CH_4_ emission inventory published in *Bousquet et al*. [2011] was used in combination with the atmospheric transport to calculate contributions from different emission sources during the previous 10 days to the CH_4_ mixing ratio at the WAS measurement points. These contributions, modeled as increments above the background, were called pseudoobservations and have been calculated using equation (4):

(4) $$\text{Increment above background}\left[{gm}^{-3}\right]=\text{Emission}\left[{gm}^{-2}s^{-1}\right]\times \text{Dilution}\left[{sm}^{-1}\right]\text{,}$$

where the dilution is calculated from the NAME footprint maps. This method is described in more detail in *Ashfold et al*. [2014].

The emission inventory used here (henceforth referred to as the modeled emissions) was created by *Bousquet et al*. [2011] and was generated using a top‐down inversion method, based on global surface measurements and a transport model. Their CH_4_ emissions have been divided into different source sectors: agriculture and waste; fossil fuel related; biomass burning; natural wetlands; all other sources, e.g., oceans and termites; and soil uptake (equivalent to a negative source). The inventory contains monthly mean emissions on a regular 1 × 1° grid between 1984 and 2009. Here the calculations were performed using each individual July monthly mean emission between 2005 and 2009, as well as the average of all of those months. The average of July 2005 to 2009 is referred to henceforth as the July emission climatology.

The wetland methane emissions from *Bousquet et al*. [2011] are similar to other inventories in recent literature. In the Scandinavian region, they fall within the range of the models in the *Melton et al*. [2013] intercomparison of wetland methane models. The total emissions north of 35°N, averaged over 1993 to 2004, in *Bousquet et al*. [2011] are 43 (±4) Tg CH_4_ yr^−1^ and 51 (±15) Tg CH_4_ yr^−1^ in the models taking part in the intercomparison. *Bruhwiler et al*. [2014] have compared their methane emissions from the CarbonTracker‐CH_4_ assimilation system and conclude that for 2007 and 2008, their results are similar to *Bousquet et al*. [2011]. Although *Bousquet et al*. [2011] is not an outlier, there is a degree of variation between the different data sets.

**Figure 6:**
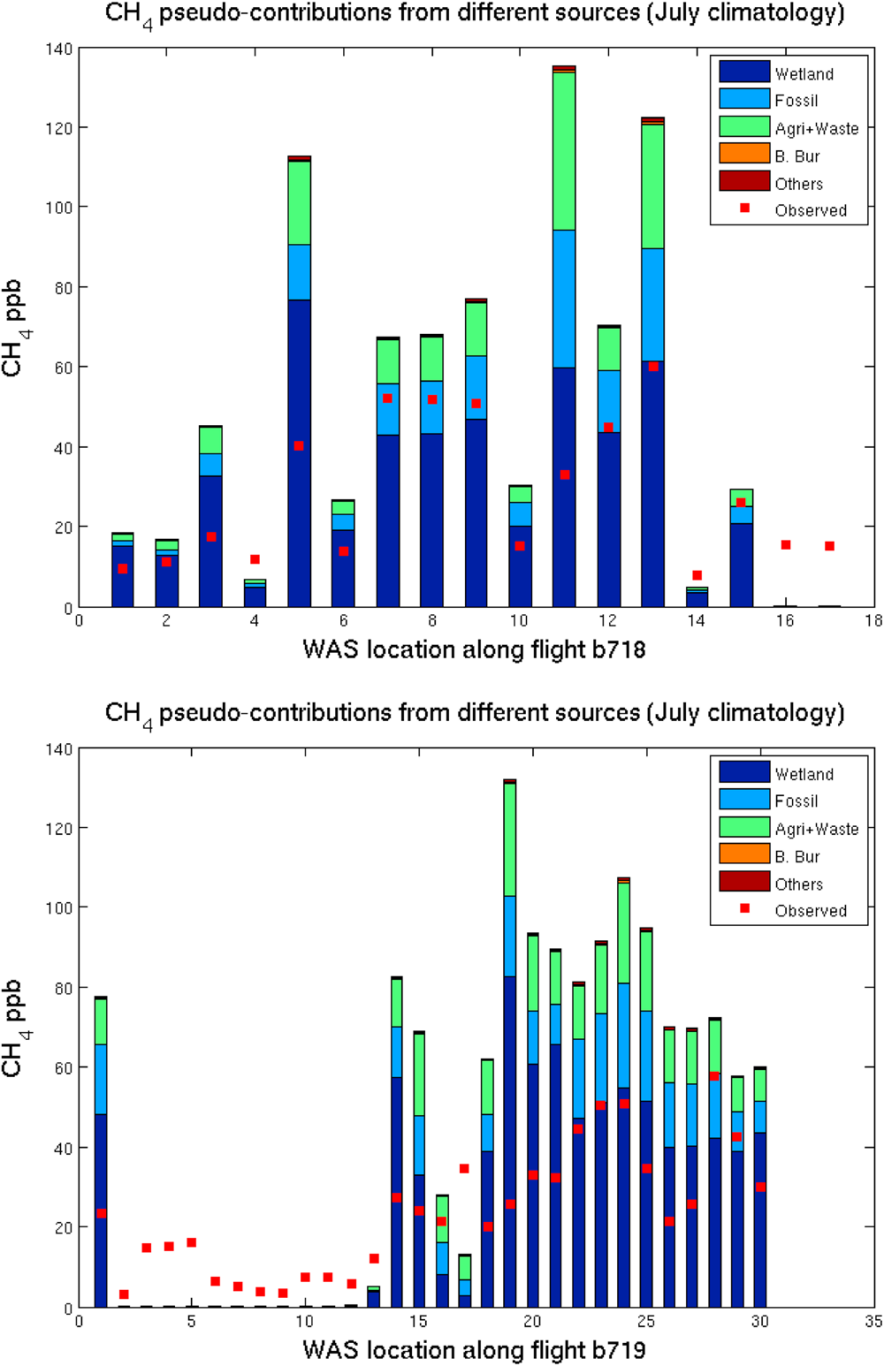
Contributions from different emission sources to CH_4_ at the WAS locations. Each bar represents a WAS location in (top) B718 and (bottom) B719. The bars are calculated by combining the July emission climatology from each source sector (wetlands, fossil fuel related, agriculture and waste, biomass burning, and others) with transport from the NAME dispersion model. The red squares show the mixing ratio from each WAS, with a background value deducted.

To quantify the contributions from the different source sectors, the modeled emissions are combined with the NAME air mass histories as described above. Figure 6 shows the contributions from each of the emission sectors considered by *Bousquet et al*. [2011] for each of the WAS locations along flights B718 (Figure 6, top) and B719 (Figure 6, bottom), using the July emission climatology. We emphasize that the CH_4_ shown represents the increment above the background that the model suggests would have been emitted from each of the source sectors. If the bar shows zero (e.g., for several WAS flasks in B719), then the CH_4_ at that location is likely to be at a background level, as the modeling suggests that it has not been influenced by surface emissions in the last 10 days. The red squares show the measured CH_4_ from the WAS with an assumed background mixing ratio deducted. The background mole fraction for each particle, dependent on its end point time and location, is taken from the MACC III CH_4_ inversion reanalysis, which has been optimized using NOAA surface observations [*Bergamaschi et al*., 2007; *Bergamaschi et al*., 2009; *Bergamaschi et al*., 2013]. The average background mole fraction of all the particles released from each WAS location was averaged to give the background value for that WAS. The WAS background values ranged between 1857 and 1866 ppb, with a mean of 1862 ppb.

The total calculated contribution to each WAS is generally greater than the observed increment above background, suggesting that either the magnitude of the modeled emission fluxes or the extent of the influence from the surface is too high at this specific time. As wetland emissions (the largest contributor, shown in dark blue) are highly interannually variable, and this calculation uses the average emission for the month of July between 2005 and 2009, it is possible that the actual emission in July 2012 was lower than this average.

**Figure 7:**
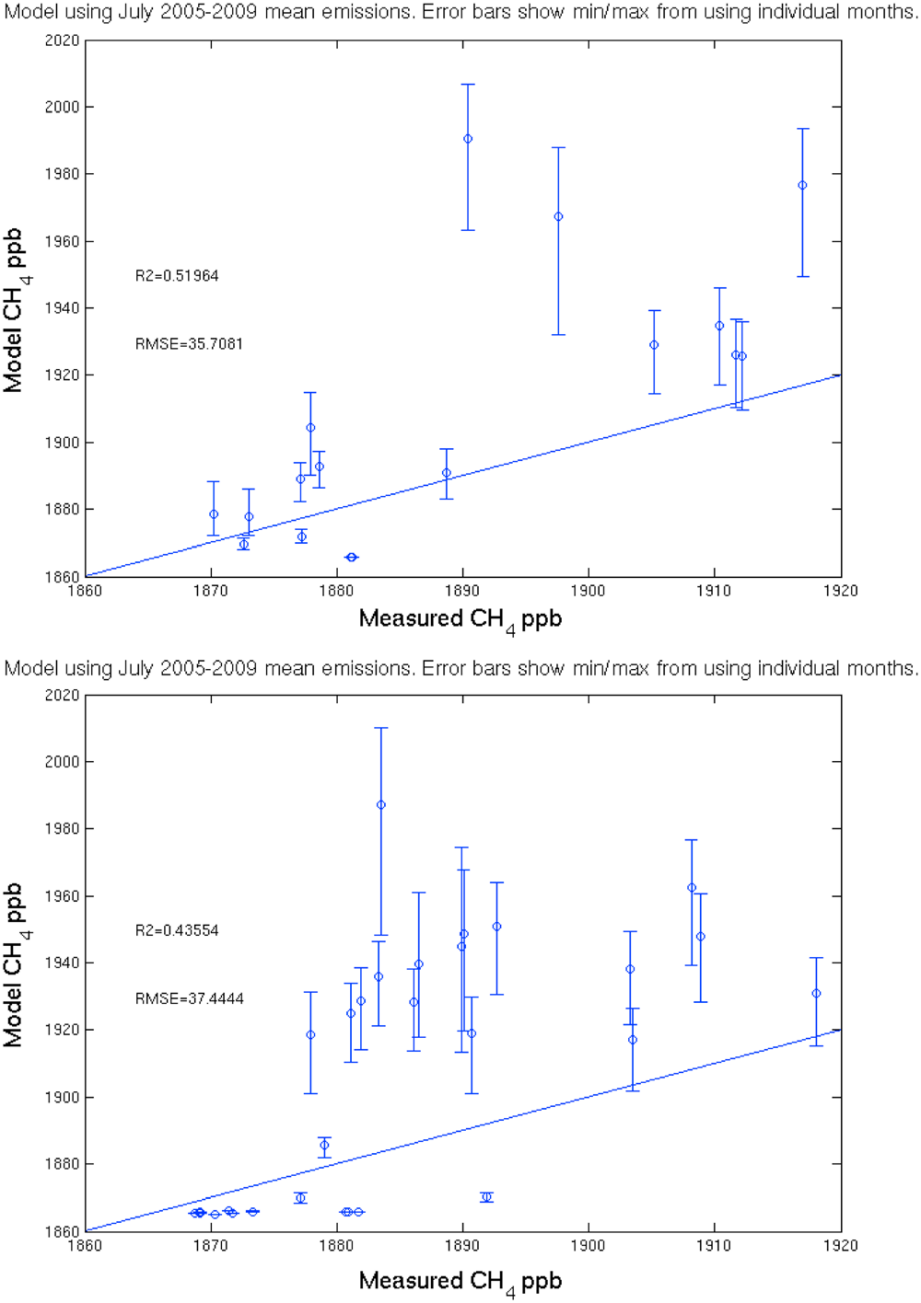
Scatterplots of the CH_4_ modeled mixing ratio (pseudoobservation) against the corresponding CH_4_ mixing ratio measured in each WAS flask for flights B718 and B719. The circles show the pseudoobservation using the July emission climatology. The whiskers show the range of values obtained by using each individual July mean emission (between 2005 and 2009). The one‐to‐one line is shown as the solid line.

The *Bousquet et al*. [2011] study only extended as far as 2009 so we cannot repeat our calculations using 2012 emissions. Instead, to assess the possible role of the interannual variability of the emissions, the calculation was repeated with emissions from each July between 2005 and 2009. Figure 7 shows a scatterplot of the CH_4_ measured from each WAS, against the calculated value (assuming a background from the MACC III reanalysis, as before). The circles show the value using the July emission climatology. The whiskers show the range between the minimum and maximum values generated using each individual month (July averages for each year between 2005 and 2009 inclusive). Consistently, July 2009 emissions show the closest agreement with the measurements, however, even they overestimate the CH_4_ increment.

Figure 6 shows that the wetland emissions alone (dark blue bars) would often overestimate the observed CH_4_ levels. Additionally, nonwetland emissions alone would in some cases also overestimate the CH_4_. This suggests that both the modeled wetland and nonwetland CH_4_ contributions are too high for this day. This could be because of uncertainties in the modeled transport and dilution or because the daily variability of the emissions is also uncertain or both.

The whiskers in Figure 7 show a large interannual variability, but any within month variability is not represented in the emission inventory. Day‐to‐day variation in wetland emission flux has been reported by *Heikkinen et al*. [2004] in a region of Russian tundra close to the Pechora River Delta. Depending on the vegetation type, their chamber measurements showed mean (standard deviation) fluxes over the season (6 June to 10 September 2001) ranging between 0.2 (±0.2) and 5.7 (±2.9) mg/m^2^/h. The range in the modeled wetland emissions used here (for monthly mean July 2005–2009) in the region of influence is approximately 1 to 2 mg/m^2^/h. The modeled CH_4_ emissions for the source region are therefore within the *Heikkinen et al*. [2004] range of observations. It could be that for this particular day, the modeled monthly mean July flux is either too high compared with the actual day's flux in magnitude or covers too widespread an area or both. To obtain a more certain result, it would be necessary to use a methane emission inventory for the specific date under investigation to take into account the meteorological influences on magnitudes of the wetland emissions. This would be beyond the scope of this study, which aims to demonstrate the application of this method using the data that are currently available to the authors.

Despite the evident overestimation, Figure 6 clearly shows that for the majority of the WAS locations, wetlands are likely the largest contributor to the CH_4_ increment, with smaller contributions from fossil fuels and agriculture and waste. This is consistent with the isotopic measurements from the WAS. In Figure 5, the Keeling plot indicates a bulk source contribution to the air mass at −70.1‰; by assigning best estimate emission δ^13^C signatures to each of the other significant sources, a value for the wetland source signature can be calculated. For the purpose of this analysis we assume that the relative contributions from the different sources in *Bousquet et al*. [2011] are robust (even though the magnitudes are overestimated compared with our observations), and then the wetland isotopic contribution can be estimated using equation (5).

(5) $$\delta^{13}C_{\text{source}}\;=\;\delta^{13}C_{x}\left[X\%\right]\;+\;\delta^{13}C_{y}\left[Y\%\right]\;+\;\delta^{13}C_{z}\left[Z\%\right]$$

where *X*, *Y*, and *Z* represent the different contributing sources of CH_4_, such as wetland, fossil fuels, and agriculture.

The overall isotopic source signature (δ^13^C_source_) is −70.1‰. We assume that a Russian fossil fuel input of −46.4 (±9)‰ [*Sherwood et al*., 2016], a combined agriculture‐waste δ^13^C signature of −65 (±5)‰, is estimated from Russian landfill data and ruminants with a C3 plant diet [*Dlugokencky et al*., 2011; *Nozhevnikova et al*., 1993], and we assume that the other sources are insignificant (Figure 6). An isotopic contribution from the wetland contribution can be calculated using equation (5) to be −78.4 (±9)‰, where uncertainties in the calculation were propagated using Monte Carlo analysis. All uncertainties are quoted to 1 standard deviation.

### 3.6 Discussion and Implications

Emission fluxes determined from mixing ratio measurements involve some kind of transport and flux inversion. Any inversion (without perfect and ubiquitous knowledge of atmospheric state) cannot deliver a unique answer, and so additional constraints need to be applied if possible. Here we have explored whether a combination of aircraft observations, Keeling analysis, and particle dispersion modeling is sufficient to determine a source region and likely source strength. Such an approach might be especially useful when the isotopic source signature suggests that measurements could have been influenced by several different sources.

For the particular case study here, there is very little doubt that the source of the elevated CH_4_ is biogenic, with a bulk Keeling analysis δ^13^C signature of ~−70‰. Given that the wetland contribution signature of −78 (±9)‰ is within the range reported from Fennoscandian wetland δ^13^C source signatures [*O'Shea et al*., 2014], it is likely to be mainly derived from a comparable wetland source. The NAME modeling indicates that a large fraction of the back trajectories at the heart of the enhanced CH_4_ air mass pass through the boundary layer over an area of northwest Russia, which is up to 20% wetland according to MODIS land use [*Friedl et al*., 2010]. However, the right plot of Figure 4 also shows that some of the back trajectories pass over the Barents Sea and Kara Sea, which could potentially be source regions for the enhanced CH_4_, which are not accounted for in the modeled emissions. The modeled emission inventory used to calculate the pseudoobservations includes only a small oceanic source from the Barents Sea, but the flux is so small that the contribution is negligible in this case (it is a component of the “others” emission source in Figure 6). Recent work has demonstrated that large areas of the Laptev Sea and Kara Sea are emitting significant amounts of CH_4_ (especially in summer months) from the thawing permafrost, with possible enhanced release from the sea to the atmosphere following storm events [*Shakhova et al*., 2014]. The Barents Sea is also shallow, largely less than 300 m in depth, and potentially could drive a similar emission system. The wind and air pressure forecast for 24 h before the flights are given in Figure S4 and show high winds in the eastern Barents Sea, which is similar to the conditions experienced by *Shakhova et al*. [2014], which resulted in the ocean CH_4_ being overturned and released to the atmosphere. It is therefore possible that the air mass seen here in this work is representative of a storm‐induced CH_4_ emission from the shallow Barents Sea, but there is currently no isotopic evidence from the Laptev Sea emissions to verify whether this has a comparable δ^13^C signature to that seen here. Methane in the seawater has been characterized near to Svalbard with δ^13^C measurements ranging from −53 to −20‰ [*Damm et al*., 2005]. Oxidation in the water column serves to enrich the heavier CH_4_ isotopes; therefore, CH_4_ in the surface waters would be heavier prior to release to the atmosphere, and −53‰ would be the lightest anticipated isotopic signature from ocean sources given currently available data. If the model attribution ratios from the NAME and inventory modeling are correct, it is difficult to reconcile an isotopically heavy fossil fuel source and a large source from surface waters while maintaining a sensible estimate for the wetland source. Therefore, the most likely main source is Russian wetlands—including land‐based or very shallow shelf permafrost degradation, essentially reactivating previously frozen wetland environments with no or little water column oxidation to drive the isotopic signature less negative. There is a clear need for greater constraints on the δ^13^C signatures of biogenic sources of CH_4_ such as thermokarst, permafrost, and hydrates, with respect to location, so that distinctive CH_4_ plumes can be traced back to their emission sources with much less uncertainty by isotope transport modeling.

Methane isotopic signatures are being increasingly used in global source and sink models to interpret trends in regional CH_4_ growth [e.g., *Monteil et al*., 2011], but as we suggest above, regionally resolved information on the pattern of δ^13^C emission signatures is poorly constrained. For example, in many instances the “generic wetland” δ^13^C signature used is above −60‰ (e.g., −59‰ [*Monteil et al*., 2011]), which is considerably heavier than the wetland inputs measured here and also during the rest of MAMM and other campaigns in Fennoscandia; a bulk signature closer to −70‰, or even lighter given the wetland component determined here (−78 (±9)‰), would seem more appropriate based on this work and others [e.g., *O'Shea et al*., 2014] and importantly makes wetland CH_4_ emissions essentially indeterminable from thermokarst lake emissions using δ^13^C ‰ [*Walter et al*., 2008]. Recommendations to improve the δ^13^C inventory for global wetlands are in line with recent work making similar revisions to the fossil fuel δ^13^C inventory [*Schwietzke et al*., 2016]. Both this work and *Schwietzke et al*. [2016] demonstrate the importance of maintaining an isotopic database for global emission studies and refining and filling in gaps in the knowledge base.

Elevated CH_4_ was also measured during sampling at the Zeppelin Observatory on Svalbard from late summer 2012, where an increase of 70 ppb CH_4_ was seen in a one off measurement of CH_4_ mixing ratio and isotopic composition on 17 September 2012. Although only a single sample and background is available to create a Keeling plot from the 2012 enhancement, the resulting isotopic source signature was −68‰ and a Hybrid Single‐Particle Lagrangian Integrated Trajectory back trajectory analysis shows a northwestern Russian/south Barents Sea source (Figure S5). Increasing the frequency of isotopic sampling of methane at Zeppelin, especially during periods of enhanced CH_4_, may resolve how frequently highly isotopically depleted emissions from Russia can reach the high Arctic.

A minimum summer bulk CH_4_ wetland (including degrading permafrost) isotopic source signature for northern Russia of −78 (±9)‰ as reported here is slightly lighter than previous work focused on the Siberian wetlands, possibly indicating that other lighter sources such as permafrost degradation are playing a role in this emission. *Tarasova et al*. [2006] reports wetland source signatures of −67.4 (±1.6)‰ for western Siberia away from industrial sources, and *Yamada et al*. [2005] report −69.8‰ for the Plotnikovo region. It is clear that the Russian wetland regions are a major atmospheric source of CH_4_, with model simulations suggesting that the Siberian wetland CH_4_ contribution to the Arctic may be considerably underestimated [*Tarasova et al*., 2009] compared to areas such as Finland. With CH_4_ mixing ratios of 2000 ppb commonly recorded north of 59°N during the daytime [*Tarasova et al*., 2006], it is easy to see how air parcels from Russia could provide large volumes of isotopically depleted CH_4_ to the Arctic troposphere.

## 4 Conclusions

We have used precision measurements of methane mixing ratios and carbon isotopes in CH_4_, together with back trajectories to determine likely emission regions for an air mass with elevated CH_4_ observed during aircraft flights in the Arctic. The signature of the total CH_4_ enhancement observed on 21 July 2012 had a δ^13^C value of −70 (±2.1)‰, with NAME back trajectories showing that the most likely sources are the northwestern Russian wetlands (including lake emissions) and coastal shelf emissions. The −70 (±2.1)‰ can be treated as a maximum (least depleted) value for δ^13^C in CH_4_ for bulk Russian natural input from this region, as any other small inputs creating a mixed signature such as burning and anthropogenic emissions (as seen in the combined NAME and inventory approach) drive the required isotopic contribution from the wetlands and coastal regions even more negative. Using the inventory and NAME modeling coupled with the isotopic data, the bulk Russian wetlands and coastal input δ^13^C signature could be as isotopically light as −78 (±9)‰. Although only a single air mass was studied, it demonstrates that large‐scale regional sources of methane are being transported over long distances to the Arctic and that the Arctic methane budget (and isotopic bulk composition) is influenced by sources thousands of kilometers away. Therefore, such extraneous sources should always be considered when interpreting Arctic methane measurements. Higher‐frequency isotopic methane sampling at stations in the European Arctic, such as at Zeppelin, would be welcome in helping to constrain the frequency and duration of such events.

Using combination techniques such as isotopes and particle dispersion modeling in tandem to sample air masses from sources that would otherwise be inaccessible demonstrates that sampling such middle‐ to long‐range air parcels in high‐latitude regions with targeted campaigns is a powerful technique to determine the influence of regional‐scale inputs. The resolution now available in particle dispersion models and inventory models, along with the ability to measure CH_4_ isotopes to high precision, will be vital to untangle not only the sources of Arctic CH_4_, but also build a larger‐scale picture of δ^13^C in CH_4_ for bulk regional emissions.

## Supporting information

Supporting Information S1
