# Supplementary material for: Measurements of δ13C in CH4 and using particle dispersion modeling to characterize sources of Arctic methane within an air mass
Source: J Geophys Res Atmos. 2016 Dec 13;121(23):14257–70. doi: 10.1002/2016JD026006 (PMC6686218; doi:10.1002/2016JD026006)
Supplement: Supplementary file 1 — Supporting Information S1 [file JGRD-121-14257-s001.docx]

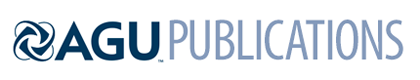


*Journal of Geophysical Research - Atmospheres*

Supporting Information for

**Identifying Sources of Long-Distance Transported Methane to the Arctic using δ^13^C in CH_4_ and Particle Dispersion Modelling.**

J. L. France^1&9^*, M. Cain^2^, R. E. Fisher^1^, D. Lowry^1^, G. Allen^3^, S. J. O’Shea^3^, S. Illingworth^3&8^, J. Pyle^2^, N. Warwick^2^, B.T. Jones^3^, M. W. Gallagher^3^, K. Bower^3^, M. Le Breton^3^, C. Percival^3^, J. Muller^3^, A.Welpott^4^, S. Bauguitte^4^, C. George^5^ , G. D. Hayman^5^, A. J. Manning^6^, C. Lund Myhre^7^, M. Lanoisellé^1^, and E. G. Nisbet^1^*.

1 {Department of Earth Sciences, Royal Holloway, University of London, Egham TW20 0EX, UK}

2 {Centre for Atmospheric Science, University of Cambridge, Cambridge CB2 1EW, UK}

3 {School of Earth, Atmospheric and Environmental Sciences, University of Manchester, Oxford Road, Manchester, M13 9PL, UK}

4 {Facility for Airborne Atmospheric Measurements (FAAM), Building 125, Cranfield University, Cranfield, Bedford, MK43 0AL, UK}

5 {Centre for Ecology & Hydrology, Maclean Building, Benson Lane, Wallingford, Oxfordshire, OX10 8BB, UK}

6 {UK Met Office, Fitzroy Road, Exeter, Devon EX1 3PB, UK}

7 {NILU - Norwegian Institute for Air Research, Dept. Atmospheric and Climate Research, Instituttveien 18, 2007 Kjeller, Norway}

8 {Faculty of Science and Engineering, MMU, John Dalton Building, Manchester, M1 5GD}

9 {School of Environmental Sciences, University of East Anglia, Norwich, NR4 7TJ, UK}

**Contents of this file**

Figures S1 to S5

**Introduction**

The supplementary figures contain supporting information to the text, but are not critical to the reading or understanding of the article.

**Supporting Information:**


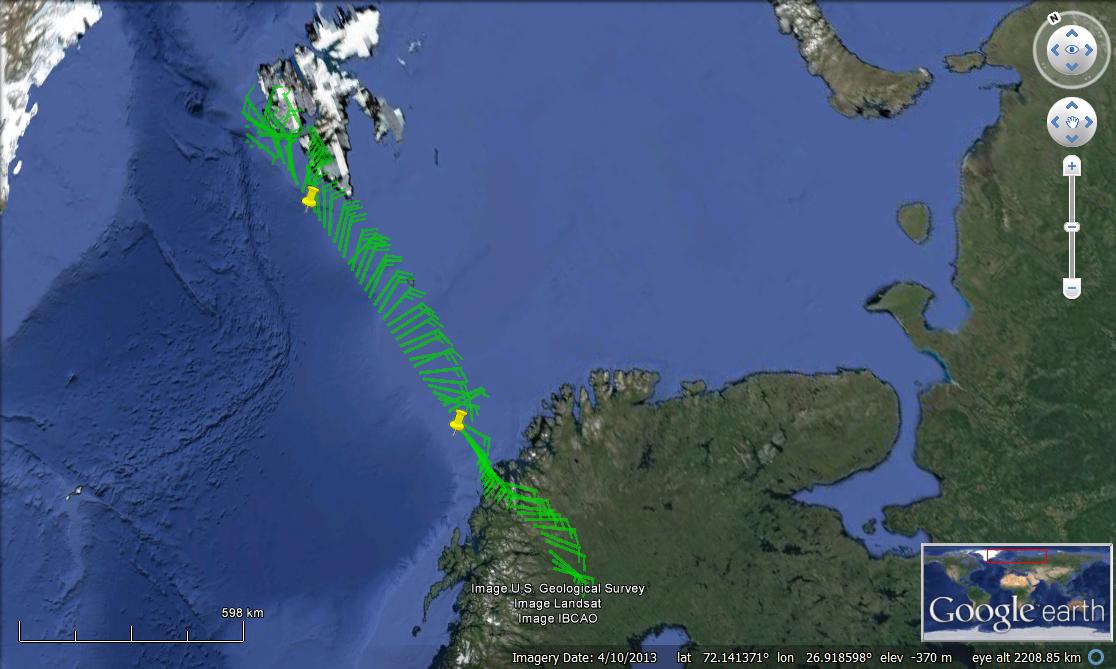


Figure S1. Airmass end points marked with yellow markers, Standard meteorological wind barbs are plotted as 3-minute average winds (measured at 1Hz frequency) from the five-port pressure measurement system on board the FAAM aircraft. Note that the wind direction is generally perpendicular to the plume transect.


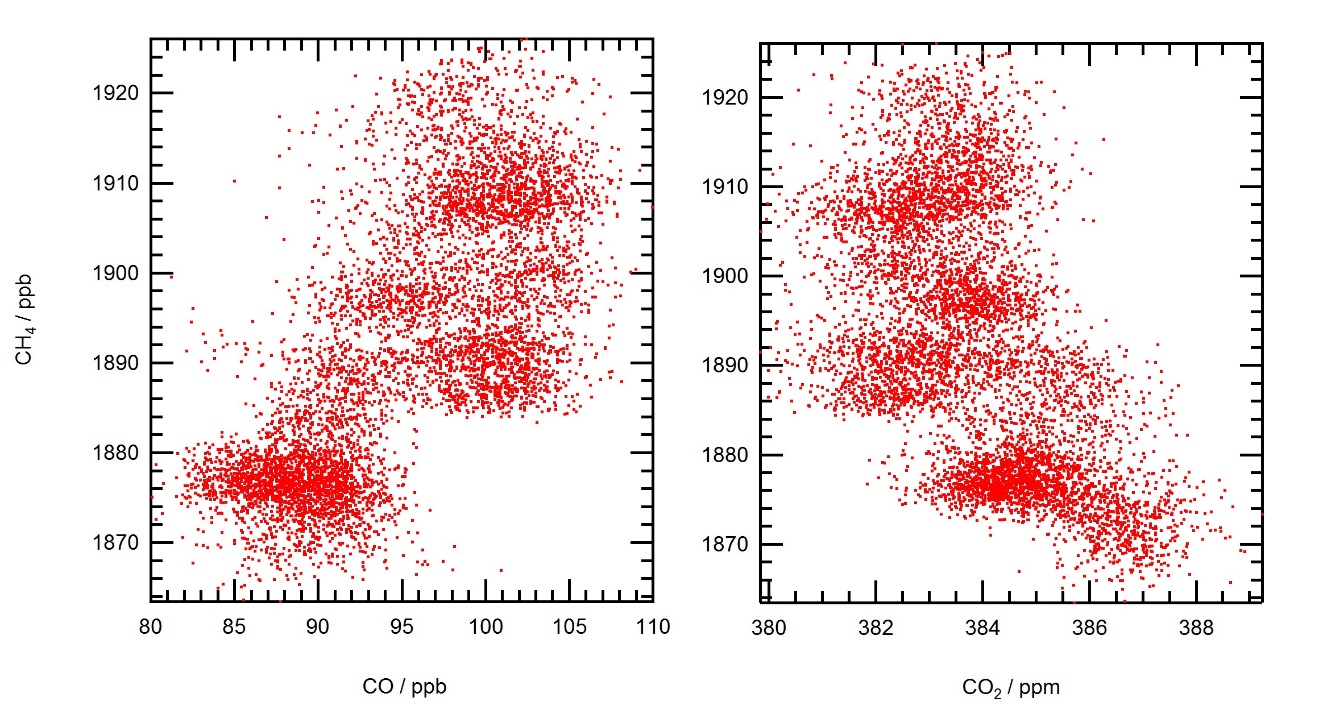


Figure S2. Scatter plots for the data shown in Figure 2. CO and CO_2_ continuous data are plotted against CH_4_ for the whole of the combined data set of the two flights.

Figure S3. HCN 30-second averaged data from the Chemical Ionisation Mass Spectrometer (CIMS) for flights B718 and B719 during the MAMM campaign on July 21^st^, 2012. This is plotted with CO (AL5002 UV fluorescence monitor) to illustrate the lack of correlation observed between CO and HCN typical of an air mass free from biomass burning.


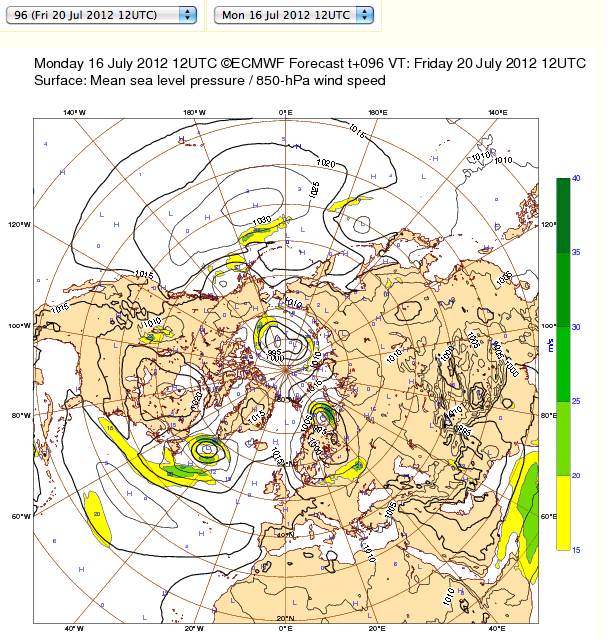


Figure S4. ECMWF Weather forecast data for the 20^th^ July 2012.


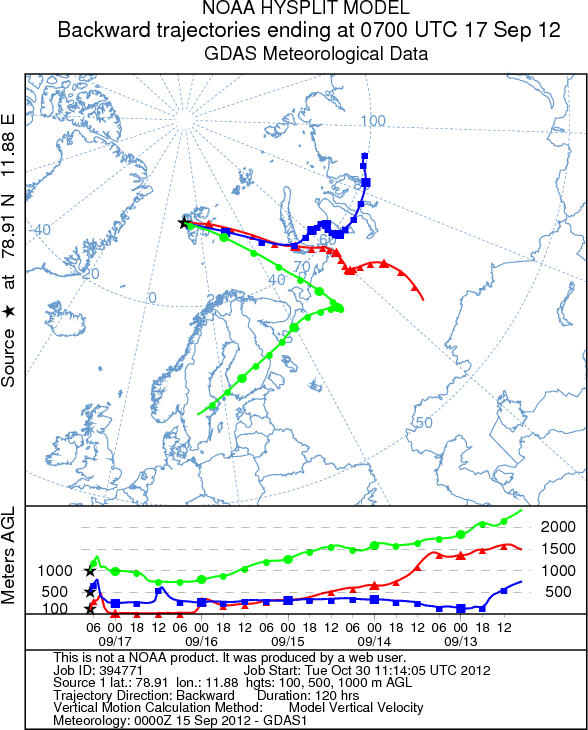


Figure S5. HYSPLIT back trajectories for 3 heights (500 m, 1000 m and 1500 m) air arrival at Zeppelin station, Spitsbergen for the 17th Sept 2012. CH_4_ were elevated by ~70 ppb above background mixing ratios on this day.
